# All Restricted Spines are not Spondyloarthritis: Fibrodysplasia Ossificans Progressiva (FOP) in Monozygotic Twins presenting to Rheumatology Clinic

**DOI:** 10.31138/mjr.210524.mta

**Published:** 2025-01-08

**Authors:** Avanish Jha, Noel Deep Luke, Aditya Nair, Ajith Sivadasan, Sumita Danda

**Affiliations:** 1 Department of Clinical Immunology and Rheumatology;; 2 Department of Clinical Genetics;; 3 Department of Neurology, Christian Medical College, Vellore, Tamil Nadu, India

**Keywords:** spines, Fibrodyplasia ossificans progressiva, ankylosing spondylitis mimic

## Abstract

Fibrodysplasia ossificans progressiva (FOP) is a rare and progressive debilitating disease that is often misdiagnosed. We present FOP in monozygotic twins in their teen years, presenting to an adult rheumatology outpatient clinic with restricted neck and spine and a referral to rule out ankylosing spondylitis. The classical feature of recurrent episodes of painful lumps on their body, along with classical deformity of their big toes and radiography, clinched the clinical diagnosis. This was further confirmed by a genetic analysis. We review here the pathogenesis and literature on newer treatment options for FOP. The first FDA-approved drug, palovarotene, was approved in 2023. It showed a reduction in heterotopic ossifications. This highlights the need for awareness of this condition among both adult and paediatric rheumatologists so that harmful biopsies and surgeries can be avoided, and patients can start on newer therapies early in the disease. It can be considered a rare mimic of ankylosing spondylitis; however, the characteristic features can very well identify the disorder clinically.

## INTRODUCTION

Fibrodysplasia ossificans progressiva (FOP), also known as myositis ossificans progressiva in the past, is a rare, debilitating, and progressive genetic disease that is often misdiagnosed.^1,2^ It has three cardinal features: malformation in toes or fingers, heterotopic endochondral ossifications, and its progressive nature, which makes individuals restricted by the 2^nd^ to 3rd decade of life.3 A child has a normal appearance and growth initially, except for the characteristic toe and finger deformities. This is followed by heterotopic ossification (HO) in the usual pattern, eventually leading to restrictions in movements.^4^ The lack of awareness about the disease and its classic manifestations leads to missed opportunities to diagnose it early. This often leads to multiple invasive biopsies and surgeries, which are known to worsen the progression of the disease.^5^ This case report aims to raise awareness regarding this disease and its promising new treatments among adult and paediatric rheumatologists as it can present to our clinics as a case of restricted neck movement.

## CASE PRESENTATION

**Figure 1.**
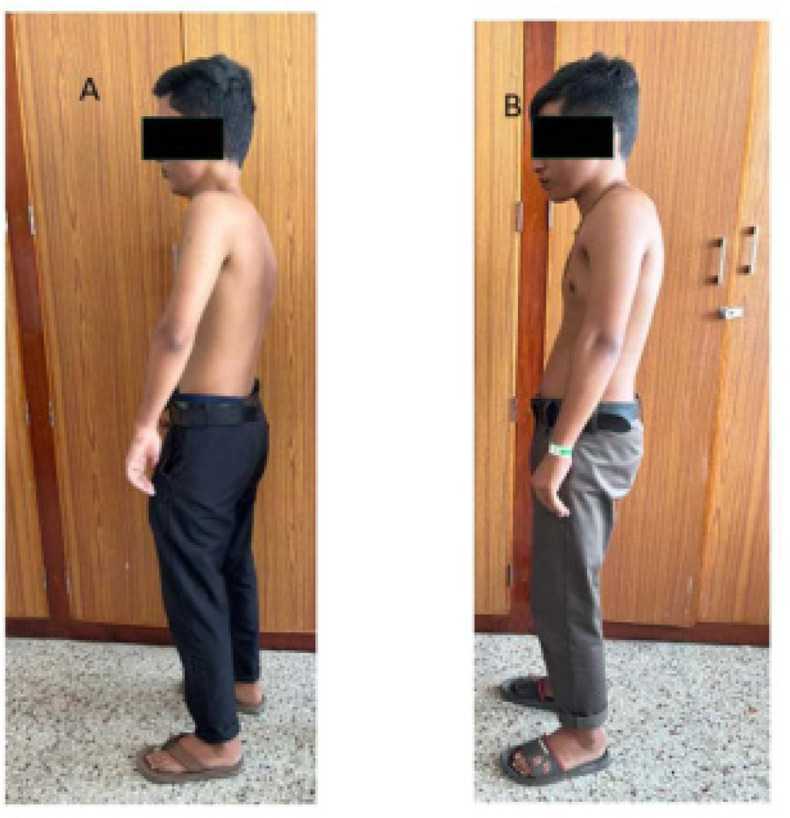
Proband A with fixed elbow and his twin brother Proband B both with noticeable stiffened posture and straightened spine.

**Figure 2.**
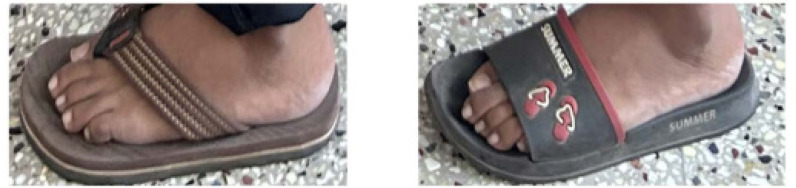
Shortened big toe of both Proband A (left) and B (right) with hallux valgus seen in Proband B.

**Figure 3.**
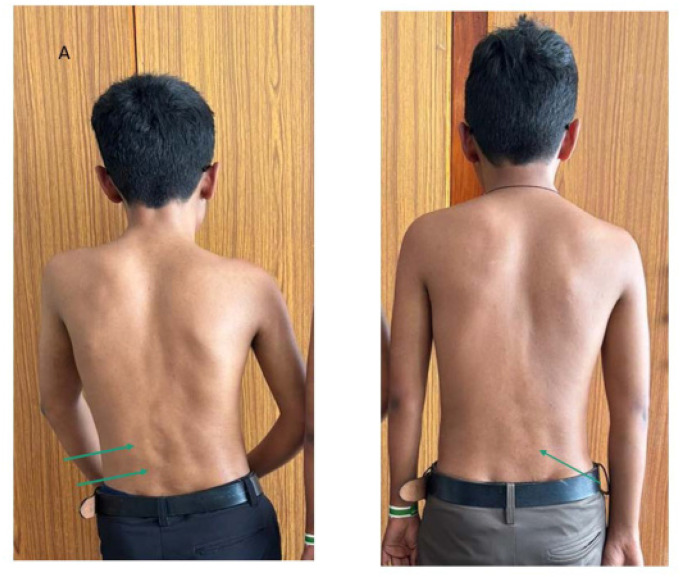
Lumbar scoliosis and fixed elbow deformities in proband A and hard discrete swellings on lower backs of both brothers are denoted by arrows.

Eighteen-year-old male identical twins (referred to as Proband A and B) presented with restricted spine movements and a referral to rule out a diagnosis of ankylosing spondylitis. Both the twins had the onset of symptoms around 14 years of age. They both had episodic onset of painful swellings on their backs and necks, and although the pains gradually settled, the swellings persisted. There were similar episodes in the upper and lower back and in the axilla. This had led to restrictions of the neck, back, and shoulders in both probands A and B by the age of 16 years. There was no history suggestive of any inflammatory back pain, peripheral joint pain, or any family history of spondyloarthritis in the twins. They were born to a non-consanguineous marriage with no other siblings. Examinations in Probands A and B revealed heights similar to their parental height but the twins weighed less than 25 percentiles for age and sex. Head-to-toe examinations were significant for both using spectacles for myopia with mildly stooped posture and dysplastic big toes with valgus deformities more obvious in proband B (**Figures 1** and **2**). Musculoskeletal examination was significant for restricted mouth opening, neck, and shoulder movements. Both had hard, painless swellings noted in the neck, axillae, and lower back. Proband A had additional findings of mild scoliosis of the lumbar spine and flexion deformities in the elbows (**Figure 3**). The rest of the systemic examination was non-contributory. The diagnoses considered were FOP, juvenile hyaline fibromatosis, and congenital myopathy with a rigid spine. Neurology and genetics opinions were taken, who considered the diagnosis of FOP in view of the classical big-toe findings.

**Figure 4.**
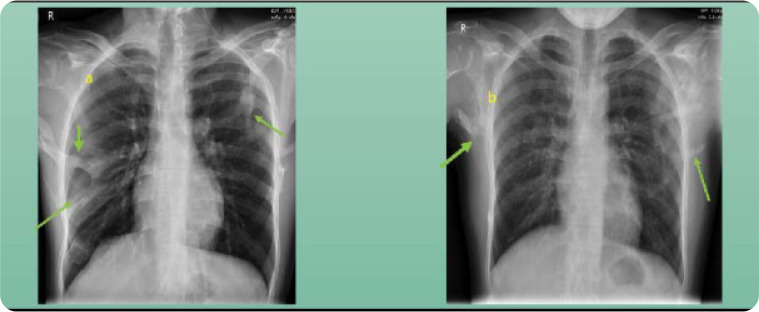
Chest radiographs from both the patients. Arrows pointing towards regions of heterotopic ossifications.

**Figure 5.**
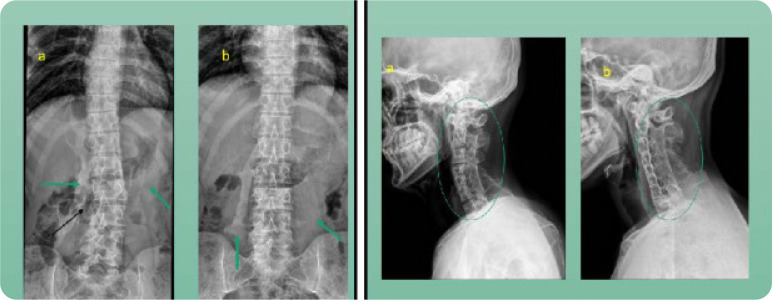
Heterotopic ossification seen in proband A and B on Lumbar AP view radiographs on the left. Lumbar scoliosis (black arrow) shown in Proband A. Loss of normal cervical lordosis, bridging ossification of posterior elements seen in both patients on the right.

The laboratory evaluation of the twins was largely un-remarkable. However, Proband A had an elevated creatine phosphokinase (CPK) to 585 units/ml. CPK was normal in Proband B. It was the radiographs that confirmed HO (**Figure 4**). The lateral view cervical spine radiograph showed loss of lordosis with ossification and fusion of facet joints with enlarged spinal processes in cervical spine. The chest and lumbar spine radiographs showed multiple areas of soft tissue ossifications (**Figure 5**).

A sanger sequencing analysis targeting a known hot-spot mutation was sent on peripheral blood from both probands A and B. *ACVR1* exon 6 c.617 G>A p. Arg206His (NM_001105.4) heterozygous pathogenic variant was found, and this confirmed the diagnosis of FOP in both brothers. This common gain-of-function mutation is present in up to 97% of all genetically diagnosed patients with FOP.^6^ They were also confirmed to be identical twins by variable number of tandem repeats (VNTR) analysis. Our patients were advised on the nature of the disease, preventive measures, and the need to maintain mobility through gentle stretching and occupational therapy. They were also advised on a demand-based treatment of acute flares with short-term corticosteroids. Etoricoxib was advised for acute flares, not needing a steroid, and other musculoskeletal pains. They were registered with fopindia.org and will be considered for novel therapies for trial or when a drug gets approval in our country.

## DISCUSSION

These two cases of FOP highlight the need for sensitisation in physicians and rheumatologists for this diagnosis, as it is often missed at the level of primary or secondary health care. FOP is an extremely rare genetic disorder with an estimated prevalence varying from 0.36 to 1.36 per million as per different studies.^7,8,9^ It is highly possible that the true prevalence is more but underreported due to patients not being attached to the registries, taking treatment under a local physician, or never being diagnosed in the community. The prevalence is, however, not estimated to be affected by race, ethnicity, or sex.^7,10^

The clinical course of this disease has a characteristic progressive pattern. The newborn most often appears normal except for the classical big toe or thumb deformities. The big toe deformities are present in almost all these patients but may be missed at birth.^3,4,9^ The most described deformities were short, big toes, lack of skin creases, and hallux valgus deformities. A short thumb may be seen in up to 65% of the cases, with a little finger clinodactyly and a broad femoral neck being the other less frequent but common features.^3^ **Table 1** describes some of the common clinical features seen in FOP. The first symptoms appear in the form of painful soft tissue swelling, usually in the first decade of life. The swelling usually appears in the axial skeleton over the cranial, dorsal, or cervical regions.^10,11^ These painful swellings may be trauma-related for half of the patients, but they can also be spontaneous. This is followed by the development of hard lumps of HO, which lead to restriction in the axial skeleton. Over 70% of individuals experienced an acute flare before the appearance of heterotopic ossification, as per one of the largest surveys of FOP patients. The flare-ups were reported to be related to viral infections, immunisations, dental or surgical procedures, and muscular trauma or overuse.^10^ Acute inflammation caused by these triggers may initiate the ossification. Mouse models have shown increased production of BMP-4 by cells of hemopoietic origin.^12^ Immune cells like mast cells, macrophages are present at site of HO.^13^ Human studies have shown elevated proinflammatory cytokines like Interleukin (IL)-3,7,8, granulocyte-macrophage-colony stimulating factor (GM-CSF) and transforming growth factor (TGF) beta in the peripheral blood of FOP patients.^14^ Monocytes from FOP patients have prolonged nuclear factor kb (NF-kb) activity and lymphocytic and mast cell infiltrates have been noted in muscle specimen from patients suggesting the role of immune cells in HO.^14,15^

**Table 1.** Common clinical features seen in Fibro-ossificans progressiva.

**Clinical Features**		**Percentage of patients with features**	**Comments**
Congenital hallux valgus malformation		97%	100% of patients with R206H mutations patients have at birth^11^
Heterotopic ossification		100%	Age dependent and episodic^11^
Inflammatory soft tissue swellings		98%	Spontaneous or triggered^22^
Scalp nodules		~40%	Maybe observed in infancy or neonatal period^41^
	Osteochondromas	~90%	Common at proximal medial tibia^22^
Other skeletal manifestations	Cervical spine fusions	~80%	Affecting C2 to C7^22^
	Scoliosis	~65%	May be rapidly progressive^11^
	Thumb malformations	~50%	Atypical or non-classical^22^
Atypical presentation		Variable	Osteochondromas of hips, degenerative joint disease of hips, sparse/thin scalp hair, mild cognitive impairment, growth retardation, etc.

The ossifications involve the appendicular regions at a later stage but usually involve the dorsal, proximal, and upper limbs more than the distal, ventral, and lower limbs. The greatest progression of heterotopic ossifications is seen in childhood and early adulthood.^16^ This addition of multiple HOs eventually causes restrictions on activities of daily living and becomes wheelchair bound by 30 years.^3,11,17^ The restriction of temporomandibular leads to poor feeding and dental hygiene and eventually being underweight.^3,11^ The cervical spine restriction that led the patients to the rheumatology clinic in our case is present in >90% of cases. The findings of enlarged posterior elements, tall, narrow vertebral bodies, and fusion of facet joints from C2 to C7 as seen in Probands A and B have been reported. These findings are strikingly like those seen in mice with homozygous deletion of noggin, a bone morphogenetic protein (BMP) antagonist.^18^ The multiple HO around the chest wall and the spine ankylosis lead to thoracic insufficiency syndrome, which leads to the risk of recurrent chest infections and poor cardiorespiratory reserve, which eventually turns fatal.^19^ This leads to a shortened median survival of 48 years in these patients.^20^ Conductive-type hearing loss, baldness, reduced reproductive capacity, and cases of mental retardation are some of the extra-musculoskeletal manifestations reported in the previous series.^3^ Our patients did not have these. Laboratory evaluations are usually normal except an elevated CPK in some cases. An elevated CPK has been correlated to the severity of traumatic HO but no such association or data is available on CPK in FOP.

FOP appears to be transmitted as a dominant trait with complete penetrance, but most often it is due to a sporadic mutation.^21^ The mutant gene was mapped to 2q23–24 using linkage analysis from five families with at least two affected members. A heterozygous mutation (617G->A; R206H) in the glycine-serine (GS) activation domain of the activin A receptor type 1 (*ACVR 1*) gene was found in all these individuals.^2^ This was also the most common mutation in previous case series reported from our centre in the past, but all these patients belonged to the paediatric age group at presentation.^22^ This remains the commonest mutation reported in FOP to date and was confirmed to be present in both our patients. There are no known *ACVR1* deletions or duplications reported to cause FOP. In cases with a strong clinical suspicion of FOP, sanger sequencing is the genetic test of choice to detect the known R206H hotspot. Alternatively, a single-gene sequencing analysis is also adequate for genetic confirmation. AVCR1 is a BMP type 1 receptor. The GS domain of ACVR1 is essential for ligand binding and is also the site of attachment for FKBP12, an inhibitory protein. Mutations in this domain cause promiscuous activation of the ACVR1 receptor by causing a conformational change which prevents FKBP12 binding and increases the ligand binding to BMPs and even activins. A dysregulated BMP signalling results in increased downstream Smad1/5/8 signalling and nuclear localisation after binding to Smad4. This causes ectopic osteogenesis and chondrogenesis.^23,24^ Inflammatory recruitment of these dysregulated pathway is evidenced by episodic flare ups. Rarer mutations in the same GS domain and the kinase domain have been reported. Both genetic mutations and environmental effects determine the phenotype, as has been seen in our patients.^25^ Both the twins had the same genotype, but Proband A had more severe involvement and appendicular involvement than Proband B. Increased paternal age has been linked to a risk of FOP in studies.^23,26^

Treatment has been the most challenging aspect of FOP disease as there were no approved therapies for this rare disease until most recently. Avoidance of trauma, surgical biopsies and intramuscular injections is mostly the preventive management advised to patients.^27^ Education of patient and caregivers, occupational therapy and stretching exercises help in rehabilitation. They can be enrolled in patient registries which can provide support and help in recruitment for trials for novel therapies. India has such a group called fopindia.org.^28^ High dose corticosteroids in dosage 2mg/kg have been used to treat acute flares involving jaw, submandibular area, or large joints as per the international clinical council FOP guidelines. This can reduce the symptoms of flare if started within 24 hours but is given for a maximum of 4 days.^29^ This is however, not proven to reduce HO. Cyclooxygenase-2 inhibitors are used after steroid is stopped or when it is used to manage the symptoms.^10^ It is not advisable to use steroid for minor flares or flares involving the back or trunk as these are usually longer duration and are recurring in nature.^27^ Mast cells are seen frequently in the pre-osseous phase of soft tissue swelling and its depletion showed improvement in HO lesions in a mouse model of FOP.^30^ Imatinib which is a C-kit inhibitor, causes mast cell apoptosis and depletion. It has been used on a compassionate basis in a few patients and has shown promising results.^31,32^ Amino bisphosphonates, with their action on bone remodelling and on inflammation mitigation, may have some role in FOP, but it’s not clear to what extent.^4^ Tofacitinib has some benefit in preventing flares as per a single centre retrospective study with 13 patients.^33^. Palovarotene became the first United States Food and Drug Administration (FDA)-approved drug for FOP for HO reduction in 2023.^34^ It lowers BMP signalling by reducing the phosphorylation of SMAD 1/5/8 proteins. It has significant adverse effects as per the phase 3 trial and needs further safety and efficacy data.^35^. Mammalian target of rapamycin (m-TOR) signalling has been shown to be important in HO, and its inhibition led to amelioration of HO in preclinical models.^36^ The clinical data is currently limited to the case reports of two patients, and they had progression of disease despite therapy.^37^ Saracatinib, a specific ACVR1 kinase inhibitor, also showed biological plausibility in a preclinical study, but clinical data is still awaited.^38^ There are other oral AVCR1 inhibitors which are being tested in phase 2 studies mentioned in **Table-2**. Garetosmab, a human anti-activin A-neutralising antibody, was used recently in a phase 2 study that didn’t meet its primary endpoint but prevented any new HO in all patients treated with it.^39^ Phase 3 of the of the study is awaited. Various other ongoing research on gene therapy via adeno-associated viruses, interfering RNA, and approaches like inhibiting glycogen synthase kinase-3 (GSK-3) beta is still in its preclinical stages.^40^

**Table 2.** Drugs and their therapeutic targets for treatment of Fibrodysplasia ossificans.

**Drug**	**Mechanism of action**	**Stage of development**	**Results**	**Comment**
**Palovarotene**	A selective retinoic acid receptor gamma agonist- inhibits phosphorylation of Smad1/5/8	Phase 3 MOVE trial completed34 US FDA approved	Mean annualised new HO volume was 60% lower in drug versus the NHS historic control	Concerns regarding early epiphyseal closure, fracture risk seen in the study Possible eye and liver toxicity to be looked for in long term follow up study
**Garetosmab**	Fully human monoclonal antibody binding and inhibiting Activin-A activation of mutated AVCR-1	Phase 2 LUMINA 1 trial phase 1 and 2 completed, phase-3 long term follow-up awaited39 OPTIMA phase 3 Trial started recruitment NCT05394116.	Phase 1- PET CT endpoint of HO activity not met Phase 2-Placebo switched over to the drug had no new HO lesions (0% in period 2 versus 40.9% in period 1; P = 0.0027)	5 deaths occurred in open label period. Causality not established Epistaxis, madarosis and skin abscesses were common adverse effects
**Saracatinib**	Oral AVCR1 kinase inhibitor	Phase 2 A- STOPFOP placebo-controlled trial undergoing recruitment in Europe42	Not posted yet No serious adverse effects in the patients who have been recruited and completed blinded period	No efficacy results available yet except from animal models Expect results in 2024
**Tofacitinib**	Oral pan Janus kinase inhibitor	Retrospective observation study in 13 cases of genetically confirmed FOP refractory to standard therapy33	Median frequency of flares decreased from 10 (6; 12) during 12 months before the baseline to 0 (0; 2) in the following 12 months and 0 (0; 0) in 24 months of treatment	Data from small single centre retrospective study Trials needed
**Imatinib**	Multiple tyrosine kinase inhibitor	2 case series published on 7 and 3 cases respectively - used on compassionate basis31,32	Improvement in intensity and number of flare ups reported in both series	No trial data available
**Fidrisertib**	Oral selective ACVR1 inhibitor	Phase 2- FALKON double blinded placebo randomised control trial- recruiting43	Not posted	Recruitment in progress for the trial
**Zilurgisertib**	Oral AVCR 1 inhibitor	Phase 2- PROGRESS placebo controlled randomised trial-started recruitment NCT05090891	Not posted	Safety acceptable as per its trial in patients with myelofibrosis

US FDA: United States of America Food and Drug Administration; HO: heterotopic ossification; NHS-National Health Service, United Kingdom; AVCR-1: Activin A receptor type I; PET-CT: Positron emission tomography-computed tomography; FOP: Fibrodysplasia ossificans.

## CONCLUSION

FOP is a rare genetic condition which should be picked up early with its characteristic malformation and diagnostic biopsies should be avoided. The diagnosis should be based on targeted sequencing of the *ACVR1* gene in presence of the clinical picture. Our cases of monozygotic twins with FOP presenting to adult rheumatology care highlights the need for sensitisation to this diagnosis and its presentation as a rare mimic of Ankylosing spondylitis. The first drug for preventing HO has been recently approved and many more new drugs are in the pipeline which may help in managing this disease in future. There is a need for prompt diagnosis to prevent any harm from diagnostic or surgical procedure and manage inflammation with routine measures till we have a more definitive management in hand. Variability in the severity of the identical twins point to environmental and epigenetic factors probably playing a role which need to be explored in future.

## CONFLICT OF INTEREST

All authors confirm no conflict of interests. Authors have full control of all primary data and agree to allow the journal to review the data if requested.

## FUNDING

No funding was used.

## ETHICAL STANDARDS

All procedures performed in studies involving human participants were in accordance with the ethical standards of the institutional research committee and with the 1964 Helsinki declaration and its later amendments. The patients have been anonymised in the case report and written consent was taken from both.

## AUTHOR CONTRIBUTIONS

AJ: Concept and research design, data acquisition and analysis, literature search, preparation of manuscript, editing and review of manuscript.

NDL: Concept and research design, data acquisition and analysis, literature search, preparation of manuscript, editing and review of manuscript.

AN: Concept and research design, preparation of manuscript, editing and review of manuscript.

AS: Data acquisition and analysis, editing and review of manuscript.

SD: Concept and research design, data acquisition and analysis, editing and review of manuscript.
